# Cardiopulmonary Remodeling In Nondiabetic Patients with Systolic Heart Failure Using Sodium-glucose Cotransporter 2 Inhibitors: An Updated Systematic Review and Meta-analysis of Randomized Clinical Trials

**DOI:** 10.2174/011573403X377983250904004839

**Published:** 2025-09-19

**Authors:** Fatemeh Chichagi, Elahe Meftah, Rahem Rahmati, Fatemeh Zarimeidani, Arian Tavasol, Kimiya Ghanbari Mardasi, Negar Omidi

**Affiliations:** 1 Cardiovascular Research Center, Tehran Heart Center, Tehran, Iran;; 2 School of Medicine, Tehran University of Medical Sciences, Tehran, Iran;; 3 Student Research Committee, Shahrekord University of Medical Sciences, Shahrekord, Iran;; 4 Student Research Committee, Shahid Beheshti University of Medical Sciences, Tehran, Iran;; 5 Students’ Scientific Research Center, Alborz University of Medical Sciences, Tehran, Iran;; 6 Tehran Heart Center, Cardiovascular Diseases Research Institute, Tehran University of Medical Sciences, Tehran, Iran

**Keywords:** Sodium-glucose cotransporter 2, (SGLT2) inhibitor, heart failure with reduced ejection fraction, (HFrEF), LV mass index, cardiac cellular volume, maximal oxygen consumption, cardiorespiratory fitness

## Abstract

**Introduction:**

Sodium-Glucose Cotransporter 2 (SGLT2) inhibitors are a class of antidiabetic drugs that have demonstrated cardiovascular risk improvement in patients with heart failure. Current evidence suggests that they can also reduce mortality, hospitalization, and renal disease progression. In this study, we aimed to evaluate the cardiac reverse remodeling potential of SGLT2 inhibitors in nondiabetic heart failure patients with reduced ejection fraction (HFrEF).

**Methods:**

We systematically searched various databases, including Web of Science, PubMed/Medline, Scopus, Cochrane, and ProQuest. After screening, five randomized controlled trials were retrieved from the initial search (8442 citations).

**Results:**

The meta-analysis revealed statistically significant and positive effects of SGLT2 inhibitors on left ventricular mass and function, cardiac matrix and cells, and cardiopulmonary fitness.

**Discussion:**

SGLT2 inhibitor users experienced a reduction in left ventricular (LV) mass (mean difference (MD): -20.06 grams, confidence interval (CI) 95%: -24.94 to -10.18, *p-*value< 0.01), LV mass index (MD: -9.79 g/m^2^, CI 95%: -13.47 to -6.11, *p-*value < 0.01), LV end diastolic volume (MD: -17.42 ml, CI 95%: -29.00 to -5.83, *p-*value < 0.01), and LV end systolic volume (MD: -17.30 ml, CI 95%: -34.35 to -0.25, *p-*value: 0.05). Correspondingly, cardiac extracellular volume (MD: -1.47, CI 95%: -2.49 to -0.46, *p-*value < 0.01), cardiac cellular volume (MD: -7.74, CI 95%: -12.30 to -3.19, *p-*value< 0.01), and cardiac matrix volume (MD: -5.33 ml, CI 95%: -8.33 to -2.33, *p-*value< 0.01) significantly decreased. Markers of cardiorespiratory fitness, including maximal oxygen consumption (VO_2_) (MD: 1.58 ml/kg/min, CI 95%: 0.60 to 2.55, *p*-value< 0.01) and the minute ventilation (VE)/carbon dioxide consumption (VCO_2_) slope (MD: -1.64, CI 95%: -3.18 to -0.09, *p-*value: 0.04), also improved. Moreover, LV ejection fraction indicated a statistically and clinically negligible rise (MD: 2.97%, CI 95%: -0.24 to 6.19, *p-*value: 0.07).

**Conclusion:**

The meta-analysis supports the potential role of SGLT2 inhibitors in enhancing LV function and reducing LV mass in HFrEF patients. These drugs can benefit HFrEF patients by improving pulmonary function and oxygenation. Treatment with SGLT2 inhibitors may be effective for outcomes associated with pulmonary and left ventricular function.

## 1 INTRODUCTION

Heart Failure (HF) is a clinical condition composed of various signs and symptoms, including fatigue, edema, and shortness of breath [1]. This disease is becoming a growing economic and health burden globally [2]. In 2019, almost 60 million people were reported to have HF [3], and nearly 108 billion USD was estimated as the global cost of HF in 2012 [4]. About 6.2 million people are suffering from HF in the United States, and 30.7 billion USD has been estimated as the cost of this disease [5]. Health service costs, medications, and missed days of work add to the overall costs of the disease, all of which indicate the need for investigating HF predisposing conditions and more effective treatment options.

Recently, several clinical trials have reported promising results for Sodium-Glucose Cotransporter 2 (SGLT2) inhibitors effectiveness on HF patients’ outcomes [6-8]. SGLT2 inhibitors (SGLT2i) are a new class of drugs classically designed for glycemic regulation by lowering glucose reabsorption in the kidney and thus increasing urinary glucose excretion [9]. It has been recommended that these medications’ diuretic effect can reduce myocardial oxygen consumption by lowering cardiac load and exert protective effects on hypertension and the cardiovascular system [10-13]. DAPA‐HF randomized controlled clinical trial has revealed that the HF patients using dapagliflozin are at a significantly lower risk of the composite of worsening heart failure or death from cardiovascular causes, as well as experiencing fewer HF symptoms, compared with the placebo group [14]. The DELIVER trial was conducted on HF patients with a wide range of ejection fraction (EF), demonstrated the ability of SGLT2 inhibitors to diminish total cardiovascular events and burden among the intervention group [15].

The subsequent studies on SGLT2 inhibitors reported positive effects on cardiac structural changes. The study by Lee *et al*. on 105 HF patients with type 2 diabetes or prediabetes reported modifications in left ventricular early systolic volume (LVESV), LV global longitudinal strain, left ventricular early diastolic volume (LVEDV), left ventricular ejection fraction (LVEF), LV mass, LV global function index, left atrial (LA) volume, and extracellular volume after 36 weeks of empagliflozin use [16]. Another study by Verma *et al*. reported changes to LV mass, LVEF, LVEDV, and LVESV after 6 months in empagliflozin users [17]. SGLT2 inhibitors’ ability to reverse LV remodeling is highly needed in HF patients with systolic dysfunction where the heart muscle is unable to contract effectively. HF with reduced EF (HFrEF, LVEF ≤40%) is a complex condition leading to insufficient organ perfusion and severe symptoms, such as shortness of breath and edema [18]. Reduced EF and HF-related LV remodeling complications are linked to high rates of mortality among patients worldwide [19]. Therefore, SGLT2 inhibitors’ ability to induce reverse remodeling can significantly benefit HFrEF patients by decreasing disease symptoms and burden.

Due to the glucose-lowering effect of SGLT2 inhibitors, patients with diabetes mellitus (DM) are commonly prescribed these medications and benefit from them. However, these medications’ effects on non-DM patients are not clear yet. EMPEROR‐Preserved and EMPEROR‐Reduced trials were unique trials that demonstrated that empagliflozin reduced the cardiovascular death or hospitalization composite among HF patients with or without DM [9, 20]. Cardiovascular outcomes improvement in HF patients following SGLT2 inhibitors use was similar regardless of DM status in the DELIVER study [15]. Moreover, a recent multi-center study revealed that SGLT2 inhibitors reduced HF-related incidents [21], highlighting that these drugs may also help patients without glycemic control problems.

SGLT2 inhibitors’ reverse remodeling potential on LV muscle, independent of their hypoglycemic effects, strengthens the importance of these drugs’ use in the management of HFrEF patients, even without DM. HF patients without blood glucose problems do not routinely take SGLT2 inhibitors; however, they can benefit from these medications through improvements in heart failure-related symptoms such as pulmonary edema and shortness of breath. Improvement in pulmonary function is a promising effect observed in SGLT2 inhibitor users, which has not been widely investigated in previous review studies [22-24]. In addition to SGLT2 inhibitors’ impacts on cardiac reverse remodeling parameters, our study's innovative aim is to also evaluate pulmonary fitness improvement in systolic HF patients without DM who used these medications.

Evidence specific to SGLT2 inhibitor effects on HFrEF patients without DM is rare. In this systematic review and meta-analysis, we aimed to evaluate the impact of SGLT2 inhibitors on established HFrEF patients without diabetes, with a focus on cardiopulmonary functional parameters, and to report the pooled quantitative effects, if applicable.

## 2 MATERIALS AND METHODS

The present study is a systematic review of various databases, including Medline/PubMed, Scopus, Web of Science, Cochrane, and ProQuest, conducted according to the Preferred Reporting Items for Systematic Review and Meta-Analysis (PRISMA) guidelines [25]. Furthermore, the study protocol has been prospectively registered in the International Prospective Registry of Systematic Reviews (PROSPERO), with the corresponding registration number CRD42024513813.

### 2.1 Literature Search Strategy

In June 2023, two authors conducted a thorough and independent search of the Web of Science, PubMed/Medline, Scopus, and ProQuest databases. They utilized a variety of keywords, abbreviations, and Medical Subject Headings (MeSH) terms such as “heart failure” OR “Systolic Failure” OR “Diastolic Failure” OR “CHF” OR “HFrEF” OR “HFpEF” OR “HFmrEF” OR “Cardiac Decompensation” OR “other synonyms” AND “SGLT2 inhibitors” OR “Dapagliflozin” OR “Gliflozin” OR “Gliflozins” OR “Empagliflozin” OR “other related words” to ensure a systematic search approach. For each keyword, we used truncation (*e.g.* heart fail*) to maximize our search sensitivity and identify any relevant study. We confined our search strategy to studies published in English, without restrictions on publication date or research design, during our initial systematic search. The detailed search strategy is accessible through the Supplementary Material Table **S1**.

### 2.2 Study Selection

After a systematic search, four other individuals used the following criteria to include the relevant studies in this review:

1. Randomized controlled clinical trial (RCTs); we utilized the methodology explained by the authors regarding participants' randomization as given in previously published protocols, the ClinicalTrial.gov website, or the study design sections for the validation of randomized controlled trials (RCTs).
2. Studies with participants diagnosed with heart failure,
3. Use of SGLT2i in the intervention group,
4. Use of placebo in the control group,
5. Participant populations included non-diabetic patients with reduced ejection fraction (EF < 40%), and
6. Imaging data, including echocardiography/cardiac magnetic resonance parameters, were reported for all participants before and after receiving the intervention/placebo.

Also, the following criteria were used to exclude irrelevant studies:

1. Review articles, observational studies, letters to editors, case reports, or book chapters,
2. Lack of an assessment of nondiabetic patients or lack of an adjustment for the diabetes status, studies on only the diabetic population,
3. Studies without a control group,
4. Comparative studies of SGLT2i with other drugs,
5. Studies with combination therapies in the intervention group, or
6. Studies on patients with acute decompensated heart failure.

### 2.3 Screening and Data Extraction

The first step was to eliminate any duplicated articles. Duplicates were automatically removed using Endnote software and then manually. Then, four co-authors reviewed the titles and abstracts and excluded irrelevant studies. After this, only studies with relevant titles and abstracts were selected for a thorough review of the full text. Upon completion of the assessment of all citations, data from the selected papers were extracted. We obtained raw data from the papers’ text, tables, or supplementary materials when necessary. As we were dealing with reverse remodeling markers, we used the mean difference (MD), which is defined as the difference between a parameter’s mean before and after an intervention. Four co-authors extracted the data, which was subsequently verified by the senior author. All discrepancies found were rectified by the senior authors (F.C, E.M, N.O).

### 2.4 Quality Assessment

The quality of the included studies was assessed by the authors using the Revised Cochrane risk-of-bias tool for randomized trials (RoB 2) criteria [26]. The RoB 2 questionnaire assesses the quality of the included studies in the following five domains: (1) randomization process, (2) intended interventions (effect of assignment to intervention), (3) missing outcome data, (4) measurement of the outcome, and (5) selection of the reported result. The corresponding author was responsible for resolving the disagreements and uncertainties [27].

### 2.5 Statistical Analysis

Data from all studies were combined to calculate the mean difference (MD) and standard deviation (SD) with an inverse variance approach and random effects model. Statistical heterogeneity was quantified by I^2^; values > 75% were considered considerable. *P* < 0.05 was considered statistically significant. Publication bias was intended to be assessed by funnel plot; however, the test was not possible due to too few included studies. A leave-one-out test was employed for sensitivity analysis when more than two papers were incorporated in the meta-analysis. We employed a random effects model for the meta-analysis, with the initial assumption assuming no disparity in remodeling components between SGLT2 inhibitor users and control groups. All included studies provided the precise number of patients involved in each outcome data analysis. We utilized the precise quantity of patients' raw reported data in our meta-analysis. The proportion of missing data was negligible in the studies analyzed (all <6%). If studies did not provide the standard deviation (SD) of change in the outcomes, it was calculated with a correlation coefficient (r) of 0.5 and the following formula from the Cochrane Handbook [28]:

SD change = √(SD^2^ pre + SD^2^ post – (2r × SD pre × SD post))

All analyses were performed using Rstudio software (version 4.3.2) with metafor and Mad packages [29].

## 3 RESULTS

**Fig. (1):**
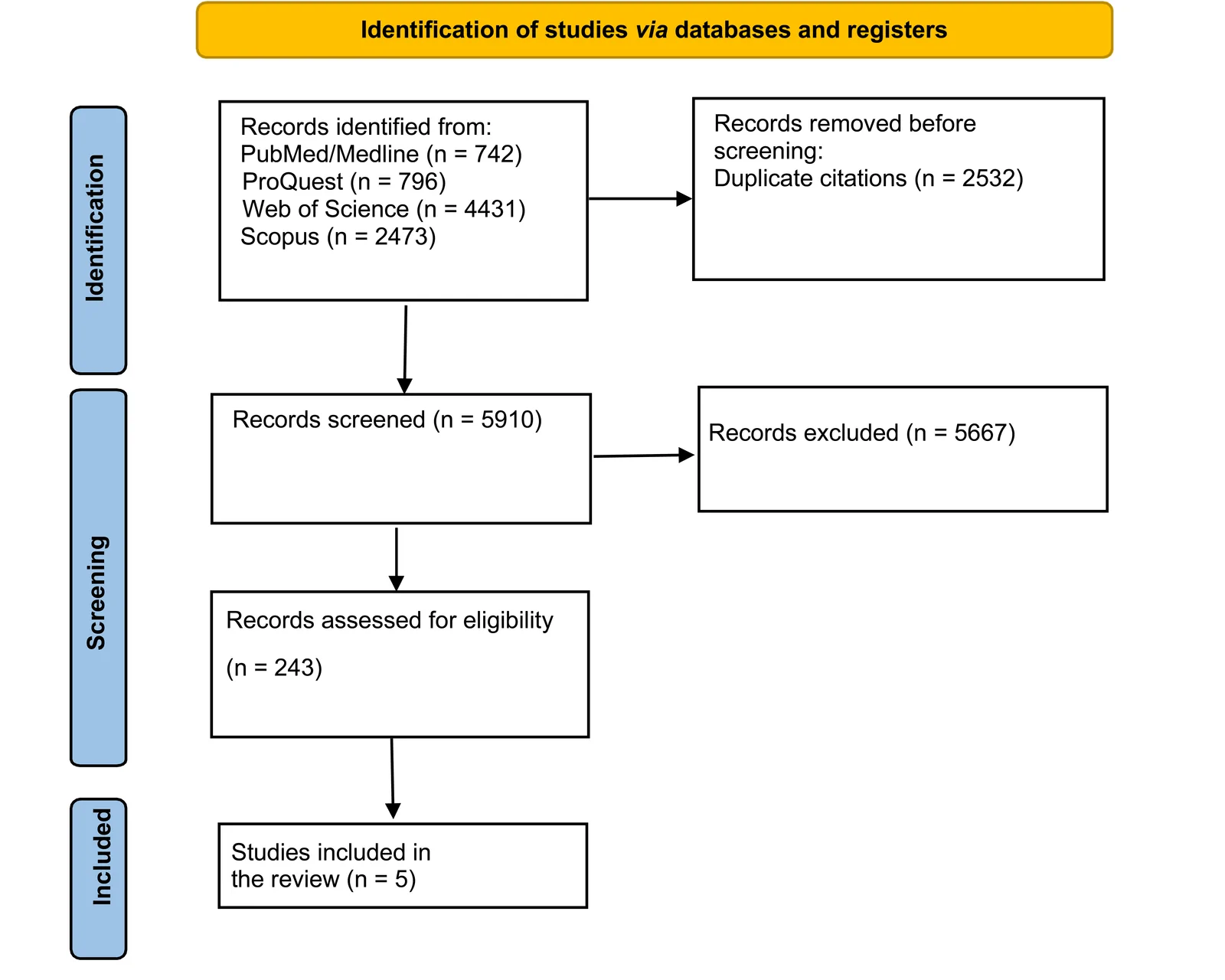
PRISMA flow diagram for systematic review.

The search results are shown in the PRISMA flow chart (Fig. **1**). The five searched databases yielded 8442 citations, of which 2532 citations were duplicates. After screening the titles and abstracts, 5667 citations were excluded, and 243 studies underwent full-text screening. Finally, five studies were included in the meta-analysis [30-34].

### 3.1 Study Characteristics and Population

Five studies with 409 participants were included in the review [30-34]. All studies used empagliflozin or dapagliflozin 10 mg per day in the intervention group, compared with placebo in the control group, and both groups were followed for 12-24 weeks. Considering the population of the included studies, only patients with heart failure with reduced ejection fraction were included in the meta-analysis. The studies’ characteristics are summarized in Table **1**.

### 3.2 Outcomes

#### 3.2.1 Left Ventricle Mass & Function

**Fig. (2):**
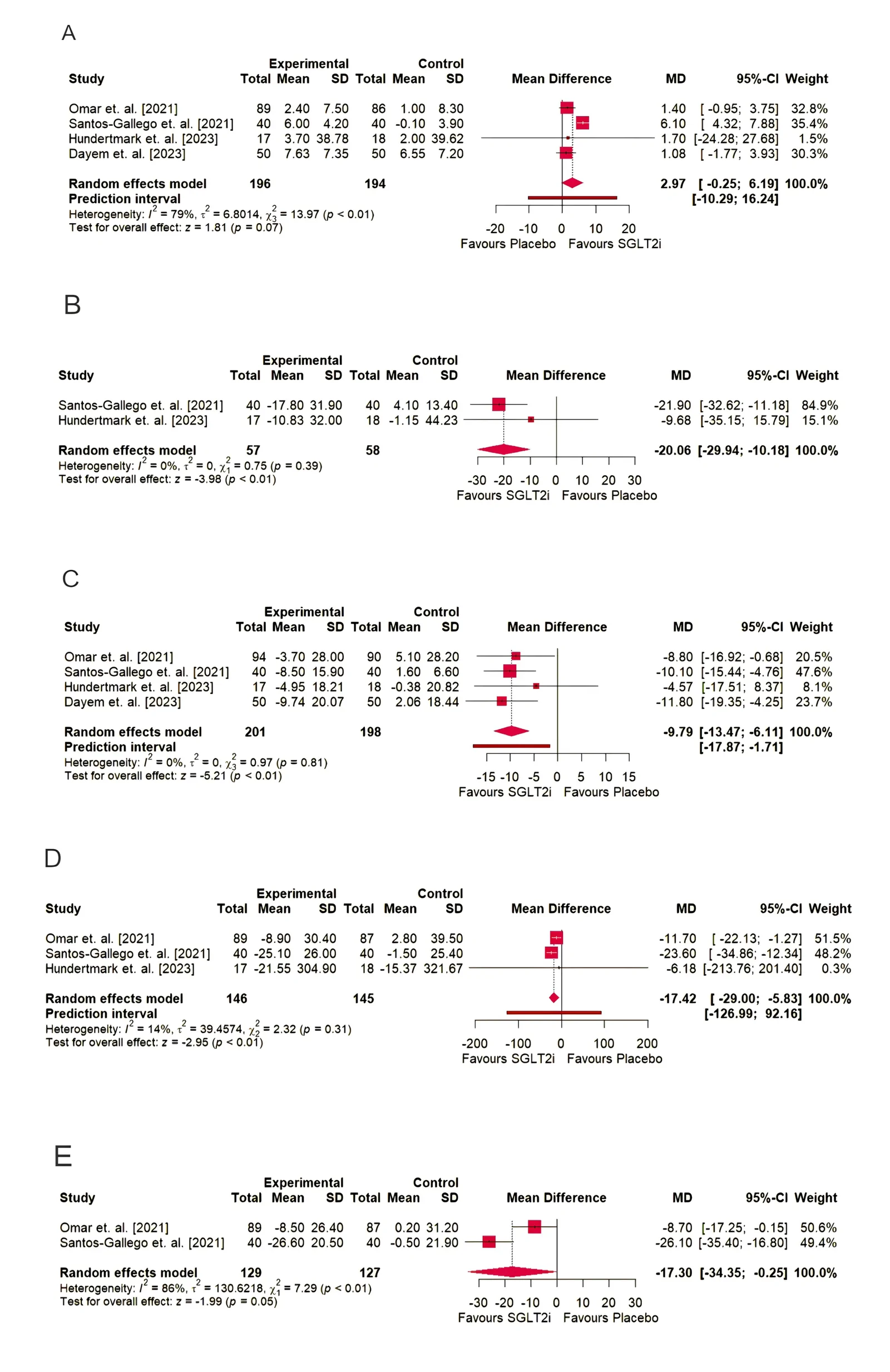
Forest plots for changes in left ventricular ejection fraction (**A**), left ventricular mass (**B**), left ventricular mass indexed to body surface area (**C**), left ventricular end-diastolic volume (**D**), and left ventricular end-systolic volume (**E**) from baseline to study endpoints in patients treated with sodium-glucose transporter-2 inhibitor therapy compared to placebo.

The hallmark of left ventricle (LV) function, LV ejection fraction (LVEF), showed a statistically insignificant increase (mean difference (MD): 2.97%, CI 95%: -0.24 to 6.19, I2: 79%, overall effect *p-*value: 0.07, four trials, Fig. **2A**).

Two indices of LV mass volume, namely LV mass (LVM) and LV mass indexed to body surface area (LVMI), were examined, both demonstrating a declining trend. LVM decreased, with a statistically significant higher drop observed in users of SGLT2 inhibitors (MD: -20.06 grams, CI 95%: -24.94 to -10.18, I^2^: 0%, overall effect *p-*value< 0.01, two trials, Fig. **2B**). LVMI also followed the same pattern (MD: -9.79 g/m^2^, CI 95%: -13.47 to -6.11, I^2^: 0%, overall effect *p-*value< 0.01, four trials, Fig. **2C**).

The administration of SGLT2 inhibitors correlated with enhancements inical trials are necessary to evaluate the iin other left ventricular functional metrics. Both left ventricular end-diastolic volume (LVEDV) and end-systolic volume (LVESV) exhibited a reduction in the SGLT2i groups relative to the placebo (LVEDV; MD:-17.42 ml, CI 95%: -29.00 to -5.83, I^2^: 14%, overall effect *p-*value< 0.01, three trials, Fig. **2D**, LVESV; MD: -17.30, CI 95%: -34.35 to -0.25, I^2^: 86%, overall effect *p-*value: 0.05, two trials, Fig. **2E**).

#### 3.2.2 Cardiac Matrix & Cell Volume

**Fig. (3):**
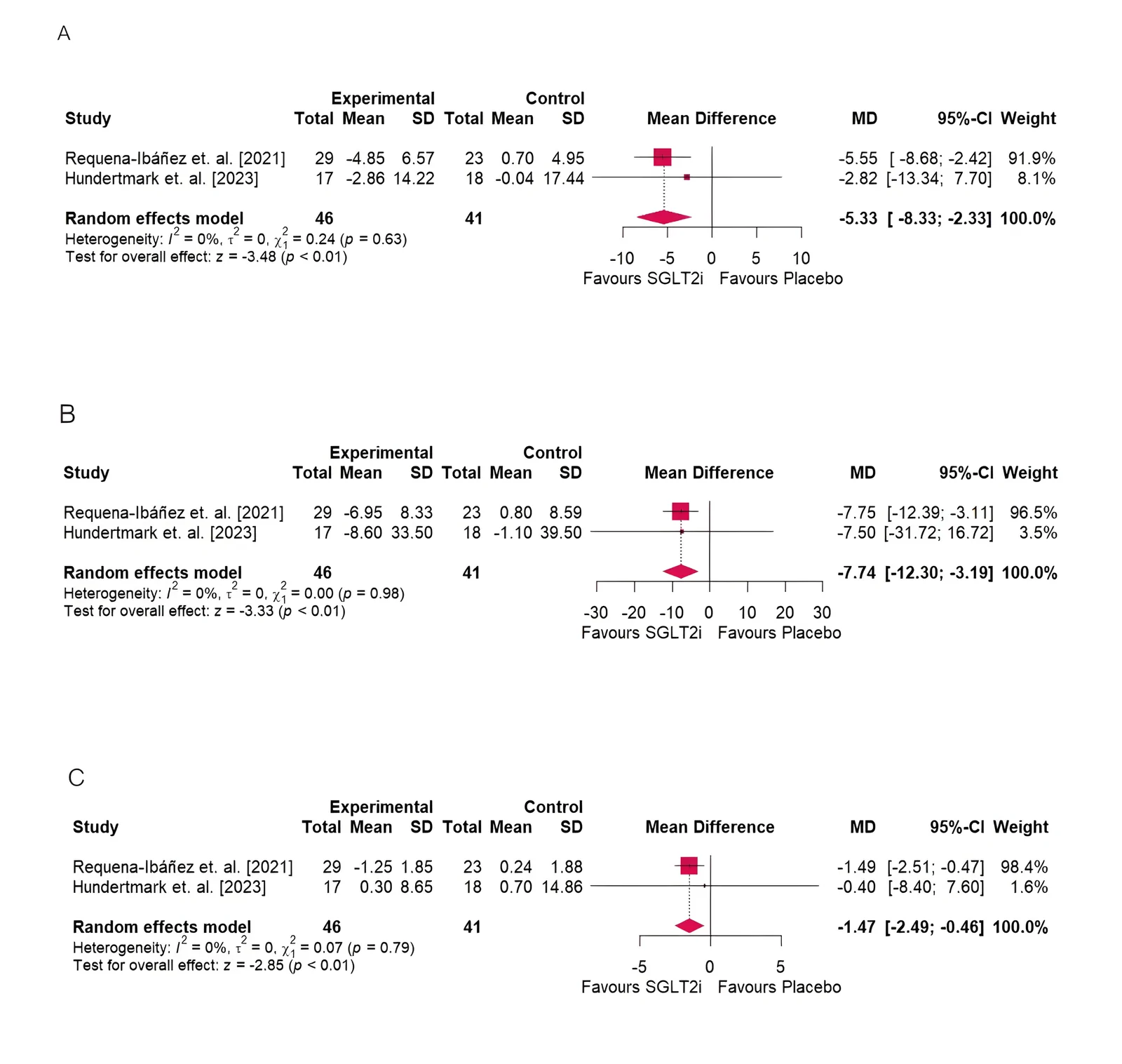
Forest plots for changes in cardiac matrix volume (**A**), cardiac cell volume (**B**), and cardiac extracellular fraction (**C**) from baseline to study endpoints in patients treated with sodium-glucose transporter-2 inhibitor therapy compared to placebo.

Cardiac matrix volume, cardiac cell volume, and cardiac extracellular volume all showed a statistically significant greater decrease in SGLT2 inhibitors users (matrix volume; MD: -5.33 ml, CI 95%: -8.33 to -2.33, I^2^: 0%, overall effect *p-*value< 0.01, Fig. **3A**, cell volume; MD: -7.74 ml, CI 95%: -12.30 to -3.19, I^2^: 0%, overall effect *p-*value< 0.01, Fig. **3B**, extracellular volume fraction (%); MD: -1.47 ml, CI 95%: -2.49 to -0.46, I^2^: 0%, overall effect *p-*value< 0.01, two trials, Fig. **3C**).

#### 3.2.3 Cardiorespiratory Fitness

**Fig. (4):**
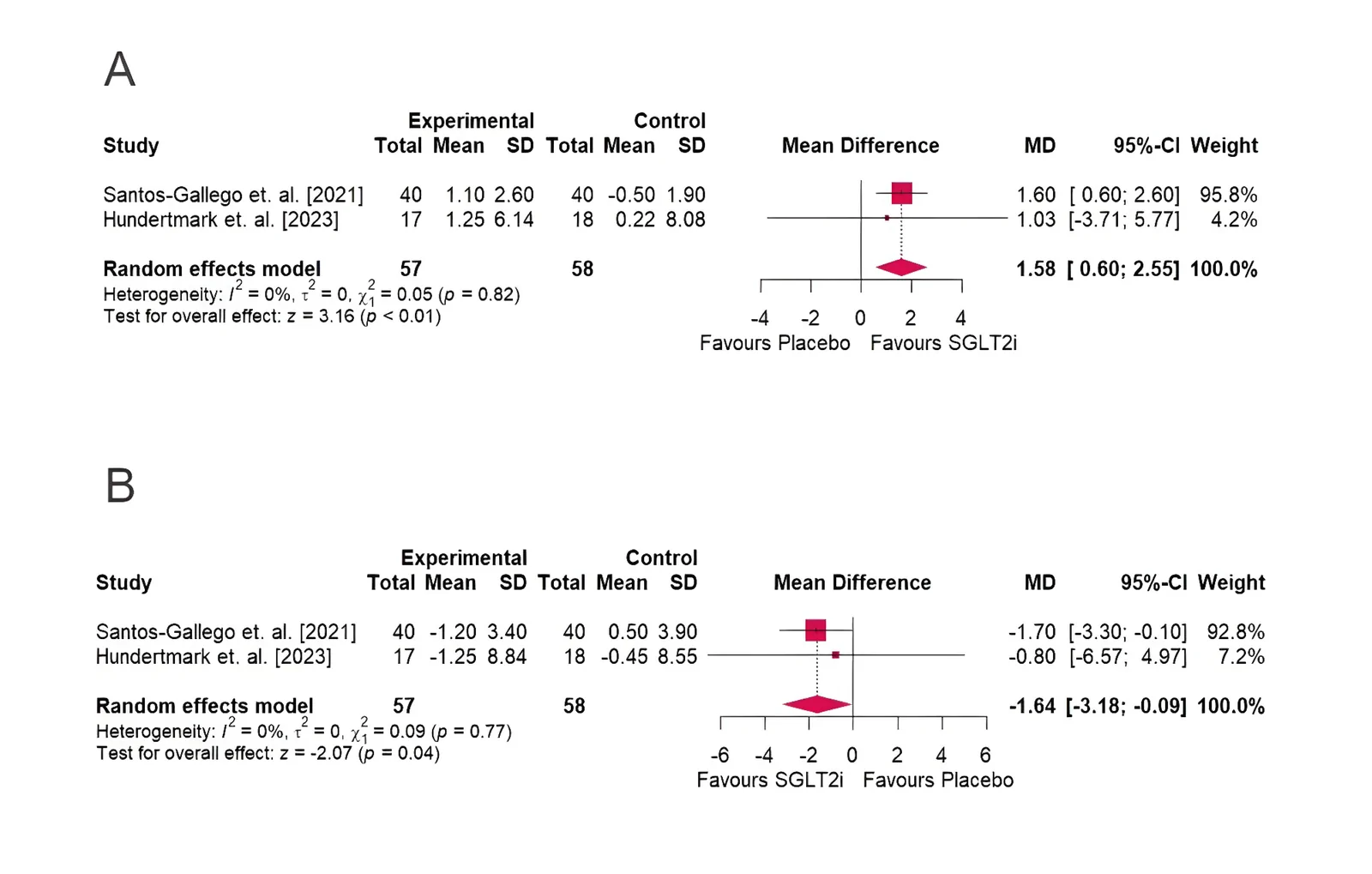
Forest plots for changes in peak VO2 (A) and VE/VCO2 slope (B) from baseline to study endpoints in patients treated with sodiumglucose transporter-2 inhibitor therapy compared to placebo. (.

Two trials evaluated cardiorespiratory tests’ changes from baseline after SGLT2 inhibitors use. After pooling results, maximal oxygen consumption (VO_2_) measures showed a higher increase in experimental group (MD: 1.58 ml/kg/min, CI 95%: 0.60 to 2.55, I^2^: 0%, overall effect *p-*value< 0.01, Fig. **4A**), and the minute ventilation/carbon dioxide production ratio (VE/VCO_2_) had a higher reduction in the experimental group compared with placebo (MD: -1.64, CI 95%: -3.18 to -0.09, I^2^: 0%, overall effect *p-*value: 0.04, Fig. **4B**).

### 3.3 Risk of Bias Assessment

**Fig. (5):**
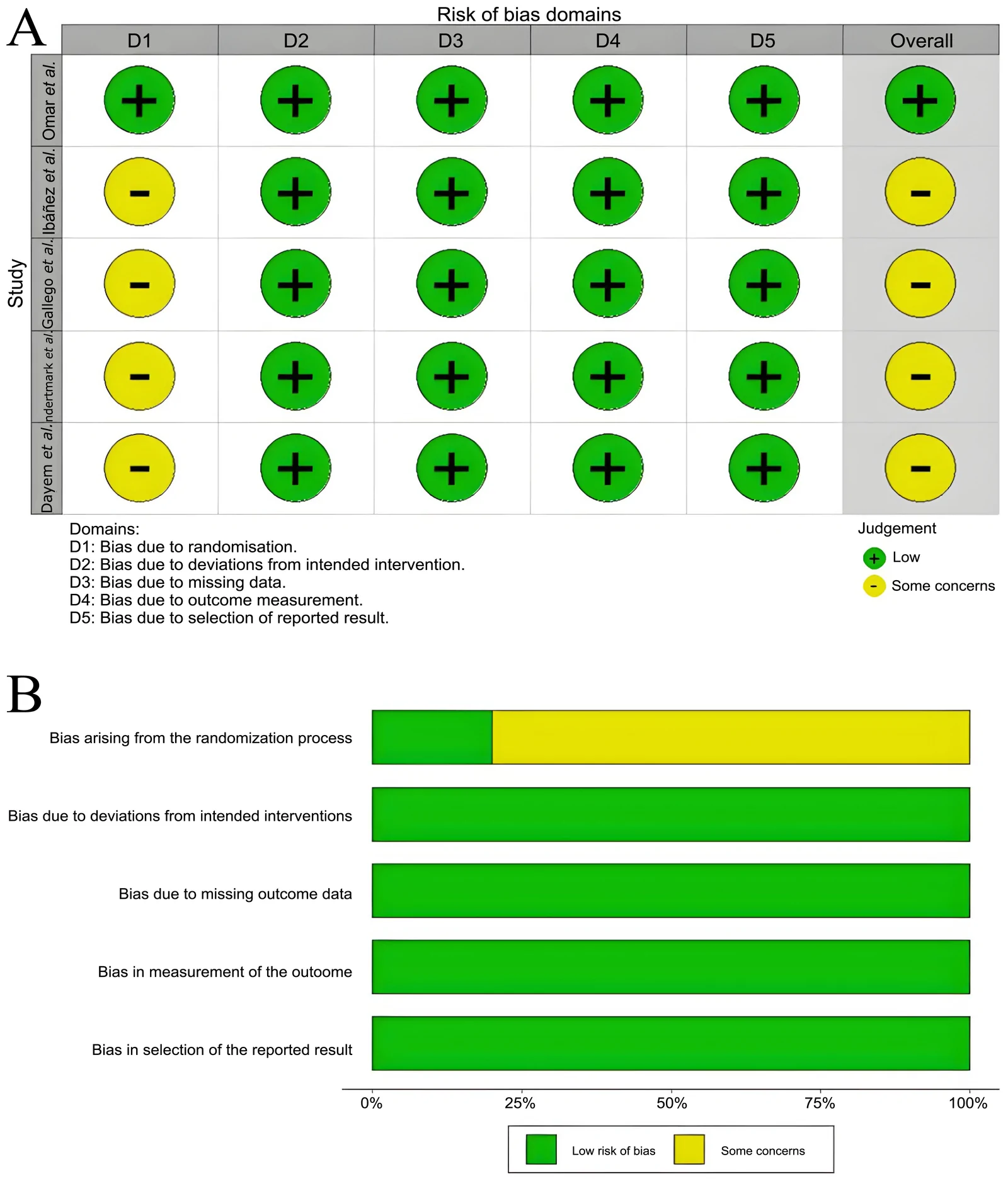
Risk of bias assessment according to the RoB 2 scoring system for A) each included study, and B) each bias domain.

The risk of bias of the included studies was evaluated using the “RoB 2” tool in five domains (Figs. **5A** and **B**). Four studies provided semi-adequate information regarding allocation concealment in the selection process, producing a moderate risk of bias in the randomization process. Other domains of bias, including deviation from intended intervention, missing outcome data, measurement of the outcomes, and selection of the reported outcomes were assessed as having a minimal risk of bias (Fig **5B**).

### 3.4 Sensitivity Analysis

**Fig. (6):**
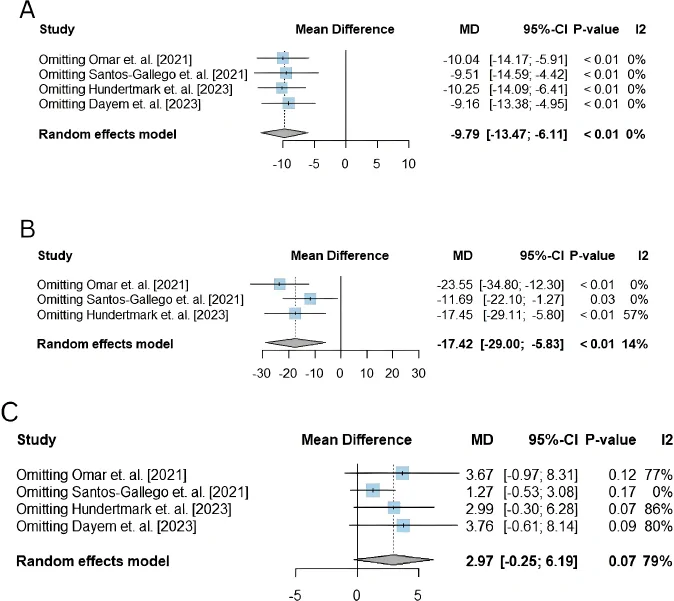
Leave-one-out sensitivity analysis for left ventricular mass index (**A**), left ventricular end-diastolic volume (**B**), left ventricular ejection fraction (**C**).

The leave-one-out test was applied to the meta-analysis that included more than two studies. Four studies were included in the pooled analysis of LVMI, and omitting each of them significantly changed the pooled effect size (Fig. **6A**, all *p*<0.01). Similarly, omitting each study from the LVED pooled effect size analysis resulted in a significant change in the pooled MD (Fig. **6B**, all *p*≤0.03). Regarding EF, no study was found to statistically change the pooled EF MD (Fig. **6C**, all *p*≥0.07).

## 4 DISCUSSION

We performed a comprehensive review of five randomized clinical trials with a total of 409 participants to investigate the effects of SGLT2 inhibitors on cardiopulmonary imaging parameters in non-diabetic heart failure patients with reduced ejection fraction. We found that SGLT2 inhibitors were effective in improving LV anatomical and functional parameters. The results demonstrated that SGLT2 inhibitors can lower LV muscle mass and volumes, including about 20 g reduction in LV mass, almost 10 g/m2 decrease in LVMI, an average of 17.5 ml reduction in LVEDV and LVESV (non-significant), and decrease in cardiac extracellular volume (1.47 ml), cell (7.74 ml), and matrix (5.33 ml) volumes. Cardiopulmonary fitness assessments revealed favorable outcomes, with a VO_2_ increase of 1.58 ml/kg/min and a VE/VCO_2_ slope reduction of 1.64. Moreover, a statistically non-significant rise of about 3% in LVEF was also noted. An insignificant increase in LVEF, approximately 3% based on our findings, is unlikely to substantially alter patients' symptoms and prognosis and is deemed clinically ineffective; however, it indicates the potential of these medications for LV remodeling, necessitating further studies to validate these results.

As heart failure progresses, the LV undergoes various changes, such as dilatation, hypertrophy, and a more spherical and balloon-shaped conformation, which damage LV structure and function and worsens the patient’s symptoms [35-37]. The remodeling process, frequently induced by myocardial infarction or chronic hemodynamic overload, alters myocardial architecture and diminishes systolic function, resulting in HFrEF [38-42]. The pathophysiology involves neurohormonal activation, inflammation, and alterations in the extracellular matrix and myo-inositol content [43], which exacerbate dysfunctional cardiac contraction and reduce cardiac output [44-47]. Clinically, these alterations present as symptoms such as exertional dyspnea, orthopnea, fatigue, and in more advanced stages, paroxysmal nocturnal dyspnea and pulmonary edema, resulting from increased filling pressures and lung congestion [48-50]. Comprehending these pathways is essential for assessing the therapeutic advantages of drugs such as SGLT2 inhibitors, which may alleviate remodeling and enhance both structural and functional results [51-53].

The evaluation of the pooled cardiac imaging parameters indicated that SGLT2i administration in heart failure patients with reduced ejection fraction had beneficial effects on reverse LV remodeling. All the included studies yielded results favoring SGLT2 inhibitors regarding LV remodeling. The study by Santos-Gallego *et al*. 2021 was conducted on 84 non-diabetic patients with HFrEF and followed them for six months. CMR results showed a greater reduction in LVEDV, LVESV, LVEF, LV mass, and LV sphericity (all *p* < 0.001) in patients who took empagliflozin 10 mg per day compared to placebo [32]. A sub-study of the Empire HF clinical trial was conducted on 190 patients with HFrEF. The experimental group was given Empagliflozin 10 mg once daily for 12 weeks, and results were adjusted for age, sex, type 2 diabetes, and atrial fibrillation. The study of Omar *et al*. resulted in the statistically significant reduction of LVESVI, LVESV, LVEDVI, LVEDV, LVMI, and Left Atrial Volume Index (LAVI) in the experimental group, all of which indicated LV reverse remodeling improvement. In this study, LVEF also improved insignificantly in the empagliflozin users [30]. Similarly, the EMPA-VISION trial by Hundertmark *et al*. showed that LVMI and LV mass decreased significantly in HFrEF patients after using empagliflozin; LVEDV and LVEF also improved insignificantly [33]. Another study by Dayem *et al*. on non-diabetic patients with reduced ejection fraction demonstrated that dapagliflozin can increase LVEF by about 8% and lower LVMI by about ten units after three months. Therefore, all included studies indicated the same direction regarding LV parameters’ changes [34]. LV volume is considered a prognostic marker for cardiovascular adverse events in HF patients, indicating the prognostic effects of SGLT2i in this group of patients [54, 55].

Previous studies indicated that SGLT2 inhibitors can reverse various pathways associated with reverse ventricular remodeling in heart failure including an increase in nitric oxide levels [42, 56-59]. Furthermore, the diuretic properties of SGLT2 inhibitors can diminish preload, hence reducing the myocardial oxygen demand for blood circulation and alleviating congestion [60-64]. It was suggested that SGLT2i might induce a metabolic shift in the myocardium by using fatty acids, ketone bodies, and branched-chain amino acids instead of glucose, which enhances myocardial energetics and improves its contractility [65, 66]. Additionally, there are additional advantageous mechanisms of SGLT2i on the myocardium that might explain the reversal of cardiac remodeling, including reduction of inflammation *via* inhibition of the nucleotide-binding domain-like receptor protein 3 (NLRP3) inflammasome activation, having downstream epigenetic-oriented effects in cardiac cells and inhibition of the mammalian target of rapamycin pathway [59, 67-70].

Cardiac fibrosis and hypertrophy represent significant remodeling responses of the heart muscle in response to heart failure [43, 71-73]. Numerous studies have demonstrated that SGLT2 inhibitors can suppress or reduce cardiomyocyte fibrosis by interacting with various signaling pathways in myocardial infarction, diabetes mellitus, and transverse aortic constriction [74-77]. Inhibition of caspase activation is an additional effect of SGLT2 inhibitors shown in mice, which can prevent remodeling in the early stages of myocardial infarction (MI) and heart failure (HF) [78-81]. Furthermore, empagliflozin has been documented to reduce excessive autophagy and prevent cardiomyocyte apoptosis [82-84]. Empagliflozin and dapagliflozin also reduce proinflammatory cytokines and oxidative stress levels, hence enhancing myocardial cell oxygenation and contraction [13, 85-87].

The pooled results of our study indicated the lowering effect of SGLT2 inhibitors on extracellular volume (ECV), cardiac matrix, and cardiomyocyte volume, which was investigated by two studies [31, 33]. Extracellular volume is a major indicator of cardiac fibrosis, which followed a decreasing pattern following SGLT2 inhibitors use in both studies. LV mass contains myocyte volume and extracellular volume; reduction in both myocyte and extracellular volume results in lower LV mass, which was confirmed by our meta-analysis results. Similarly, a recent study reported ECV reduction in diabetic patients after empagliflozin use, pointing to the SGLT2i potential for regression of myocardial fibrosis [88]. SGLT2 inhibitors can affect cardiac fibrosis through glycemic and non-glycemic pathways. High levels of blood glucose can lead to the activation of cardiac fibroblasts and cytokine overproduction, which accumulate in the cardiac interstitium and blood vessels. This effect is preventable by the glucose-lowering impact of SGLT2 inhibitors. Also, these drugs have regulatory effects, including anti-inflammation and anti-oxidation, which can combat cardiac fibrosis by regulating the TGF-β signaling pathway [89, 90].

Chronic systolic dysfunction may lead to significant pulmonary problems. Left heart failure primarily results in pulmonary edema. The lung becomes rigid and dense due to elevated collagen levels and thickened alveolar walls, disrupting oxygen exchange [91]. Pulmonary functional capacity status has been reported as a prognostic factor in HF [92, 93]. Due to HF-induced pulmonary dysfunction, the current guidelines advocate for cardiopulmonary exercise testing in heart failure patients to assess functional capacity and therapy suitability, owing to significant pulmonary symptoms [94]. Our results demonstrated that SGLT2 inhibitor consumption raised VO_2_ maximum and lowered VE/VCO_2_ slope. Two trials investigated the mentioned cardiopulmonary fitness tests and resulted in improved VO_2_ and decreased VE/VCO_2_ slope [32, 33]. Similarly, previous studies showed positive effects of SGLT2 inhibitors on cardiopulmonary tests. A meta-analysis of 17 studies also demonstrated a 1.6 ml/kg/min improvement in VO_2_ in heart failure patients after using SGLT2 inhibitors [95]. The EMBRACE-HF trial indicated empagliflozin's substantial decrease in pulmonary artery pressure among heart failure patients. The usage of Canagliflozin has been previously associated with a reduction in the thickness of pulmonary arterioles' walls *via* inhibiting the growth of smooth muscle cells [96]. Given the extensive pulmonary symptoms in heart failure patients with systolic dysfunction, medications that enhance pulmonary function and may facilitate lung remodeling are very pertinent for consideration. Nevertheless, the data remains insufficient, necessitating future randomized trials, especially in patients with HFrEF and pulmonary dysfunction.

Previous investigations indicate that SGLT-2 inhibitors are efficacious in the pulmonary endothelium, smooth muscle cells, and arterial remodeling. Animal studies demonstrated that SGLT-2 inhibitors might diminish oxidative stress (reactive oxygen species formation in mitochondria) and inflammatory responses (interleukin (IL)-1, IL-6, tumor necrosis factor-α), while restoring endothelium-dependent vasodilation in both diabetic and non-diabetic conditions [97, 98]. Inhibition of SGLT2 receptors is associated with enhanced nitric oxide bioavailability, increased platelet-derived growth factor, activation of epidermal growth factor receptors, and stimulation of the eNOS signaling pathway, resulting in dilation of pulmonary arteries, angiogenesis, and improved exercise tolerance [93, 99, 100]. Given the efficacy of these drugs on inflammation and angiogenesis, SGLT2 inhibitors are anticipated to ameliorate pulmonary hypertension and fibrosis. The potential of SGLT2 inhibitors for the treatment of pulmonary diseases has not been extensively studied and requires further randomized clinical trials.

Our findings revealed significant heterogeneity across the included studies (I^2^: 79%, *p*<0.01 for pooled LVEF effects; I^2^: 86%, *p*<0.01 for pooled LVESV effect), indicating considerable diversity in the reported outcomes. This diversity may stem from the differing characteristics of the populations in the included studies.

The participants in the study by Omar *et al*. were predominantly white (98%), whereas the trial conducted by Santos-Gallego *et al*. included a diverse racial composition, with just 27% of participants identifying as white [101]. Santos-Gallego *et al*. published data after six months of SGLT2 inhibitor treatment, which exceeded the duration of the investigations conducted by Omar *et al*. [30] and Hundertmark *et al*. [33]. Furthermore, several parameters influencing left ventricular remodeling, such as the duration of heart failure, were not reported in several studies. Given the restricted number of participants in each study, minor variations in population characteristics can result in statistically significant discrepancies in the outcomes and produce high heterogeneity. Similarly, the limited number of studies heavily affected the pooled results, and omitting each of the included studies can significantly change the reported pooled effect size (Fig. **6**). Additional randomized clinical trials across diverse populations are necessary to arrive at a definitive conclusion regarding the effects of SGLT2 inhibitors.

Furthermore, it is essential to note that the follow-up duration of the included studies ranged from 12 to 24 weeks, which is a relatively brief period for assessing reverse remodeling. This period is enough for identifying initial enhancements, such as decreases in left ventricular sizes and alterations in myocardial tissue composition, but it may not comprehensively reflect the persistence or long-term magnitude of structural cardiac modifications. Data from cardiac resynchronization therapy trials demonstrate that reverse remodeling frequently persists beyond six months, with certain effects reaching their zenith at two years and enduring for as long as five years [92, 93]. Long-term findings on SGLT2 inhibitors indicate that ongoing benefits may manifest over time, underscoring the importance of prolonged follow-up to assess the durability and therapeutic significance of initial structural enhancements [102-104]. Our results did not demonstrate the therapeutic clinical effectiveness of SGLT2 inhibitors; however, a statistically significant difference was seen between the intervention and control groups. A small increase in EF or a decrease in cardiac matrix or cell volume cannot exert significant impacts on the patients’ symptoms. Future trials in large populations of patients with extended follow-up durations are necessary to ascertain the precise effects of these drugs on cardiopulmonary symptom improvement and patients’ quality of life.

## CONCLUSION

This present study reinforces the significant beneficial effects of SGLT2 inhibitors on cardiac and pulmonary function and structural improvement in HFrEF patients. Hence, SGLT2 inhibitors administration in these groups of patients, regardless of their glycemic status, is suggested due to their effects on reversing LV remodeling indices, and possibly reducing morbidity and mortality. Furthermore, additional clinical trials are necessary to evaluate the impact of these drugs on non-diabetic individuals, particularly those with cardiopulmonary dysfunction. The impact of SGLT2 inhibitors on pulmonary vascular remodeling has not been extensively studied. Future investigations should concentrate on the drugs' potential as an emerging therapy for pulmonary hypertension and fibrosis. Furthermore, the studies included predominantly brief follow-up durations (< 6 months), which are inadequate for assessing cardiopulmonary remodeling indices. Additional randomized clinical trials with extended follow-up are necessary to evaluate the efficacy of SGLT2 inhibitors for cardiorespiratory remodeling in individuals with HFrEF, irrespective of diabetes mellitus status.

## STUDY LIMITATIONS

Our study has several limitations. There was a difference in the duration of follow-up and some baseline characteristics. Diverse baseline characteristics with varying confounding effects are a source of bias in all observational studies. The follow-up duration was notably brief and varied among the included studies. Additionally, the limited number of included studies, as well as participants, significantly affected the heterogeneity and the practical value of the meta-analysis and prevented us from conducting meta-regression analysis. Considering the limited number of clinical trials evaluating SGLT2 inhibitors' effects on non-diabetic patients with HFrEF, most of the analyses combined only two trial results, which can severely affect the meta-analysis results and weaken the analysis power. Some studies included in the meta-analysis published crude hazard ratios that did not address the potential confounders; this also can affect the results. Therefore, we should be cautious in interpreting the results and work on more clinical studies investigating the effectiveness of SGLT2i on cardiorespiratory remodeling in HFrEF patients in the future.

## Figures and Tables

**Table 1:** Population characteristics of the included studies.

**Study Author**	**Study Design**	**Number**	**Mean Age (Years)**	**Participants** **(Men)**	**Non-Diabetic Patients**	**NYHA Class**	**Intervention *vs* Controls**	**Outcomes of Interest**	**Follow-up Time (Months)**
Omar *et al*. (2021) [30]	RCT	190	64±11	162 (85.3%)	166 (87.3%)	I to III	Empagliflozin 10mg daily or matching placebo in addition to recommended heart failure therapy.	LVESV, LVEDV, LVEF, and LVMI	3
Requena-Ibáñez *et al*. (2021) [31]	RCT	84	62±12.1	54 (64%)	84 (100%)	II & III	Empagliflozin 10 mg daily or matching placebo.	Extracellular volume fraction, matrix volume, and cardiomyocyte volume	6
Santos-Gallego *et al*. (2021) [32]	RCT	84	62±12.1	54 (64%)	84 (100%)	II & III	Empagliflozin 10 mg daily or matching placebo.	LVEDV, LVESV, LV mass, LVEF, peak VO_2_, and VE/VCO_2_ slope	6
Hundertmark *et al*. (2023) [33]	RCT	35	67.90±13.8	23 (65.7%)	30 (85.7%)	II to IV	Empagliflozin 10 mg daily or matching placebo.	LVEF, LVEDV, LV mass, LVMI, extracellular volume fraction, cell volume, matrix volume, peak VO2, and VE/VCO_2_ slope	3
Dayem *et al*. (2023) [34]	RCT	100	55.97±12.3	83 (83%)	100 (100%)	NA	Dapagliflozin 10 mg, daily, or matching placebo.	LVEF, and LVMI	6

**Abbreviations:** LV: left ventricular, LVESV: left ventricular end systolic volume, LVEDV: left ventricular end diastolic volume, LVEF: left ventricular ejection fraction, LVMI: left ventricular mass index, peak VO2: maximal oxygen consumption, VE/VCO_2_: minute ventilation/carbon dioxide production, RCT: randomized clinical trial, NYHA: New York Heart Association.

## Data Availability

All the data and supportive information are provided within the article.

## LIST OF ABBREVIATIONS

HF: Heart Failure
LA: Left Atrial
LVEDV: Left Ventricular Early Diastolic Volume
LVEF: Left Ventricular Ejection Fraction
LVESV: Left Ventricular Early Systolic Volume
MD: Mean Difference
RCTs: Randomized Controlled Clinical Trial
SD: Standard Deviation
SGLT2: Sodium-Glucose Cotransporter 2

## STANDARDS OF REPORTING

PRISMA guidelines and methodology were followed.

## SUPPLEMENTRY MATERIAL

PRISMA checklist is available as supplementary material on the publisher’s website along with the published article.
